# Patient and public co‐creation of healthcare safety and healthcare system resilience: The case of COVID‐19

**DOI:** 10.1111/hex.13659

**Published:** 2023-05-04

**Authors:** Abigail Albutt, Lauren Ramsey, Beth Fylan, Chloe Grindey, Isabel Hague, Jane K. O'Hara

**Affiliations:** ^1^ Yorkshire Quality and Safety Research Group Bradford Institute for Health Research, Bradford Teaching Hospitals Bradford UK; ^2^ School of Pharmacy and Medical Sciences University of Bradford Bradford UK; ^3^ School of Healthcare University of Leeds Leeds UK

**Keywords:** COVID‐19 pandemic, qualitative study, resilient healthcare theory

## Abstract

**Introduction:**

Healthcare system resilience is a conceptual approach that seeks to explore how health services adapt and respond to variability in demand and resources. As has been witnessed since the beginning of the COVID‐19 pandemic, healthcare services have undergone many reconfigurations. One understudied aspect of how the ‘system’ is able to adapt and respond is the contribution of key stakeholders—patients and families, and in the context of the pandemic, the general public as a whole. This study aimed to understand what people were doing during the first wave of the pandemic to protect the safety of their health, and the health of others from COVID‐19, and the resilience of the healthcare system.

**Methods:**

Social media (Twitter) was used as a method of recruitment due to its ability for social reach. Twenty‐one participants took part in 57 semistructured interviews over three time points from June to September 2020. The included an initial interview and invitation to two follow‐up interviews after 3 and 6 weeks. Interviews were conducted virtually using Zoom—an encrypted secure video conferencing software. A reflexive thematic analysis approach to analysis was used.

**Results:**

Three themes, each with its own subthemes were identified in the analysis: (1) A ‘new safety normal’; (2) Existing vulnerabilities and heightened safety and (3) Are we all in this together?

**Conclusion:**

This study found that the public had a role in supporting the resilience of healthcare services and systems during the first wave of the pandemic by adapting their behaviour to protect themselves and others, and to avoid overwhelming the National Health Service. People who had existing vulnerabilities were more likely to experience safety gaps in their care, and be required to step in to support their safety, despite it being more difficult for them to do so. It may be that the most vulnerable were previously required to do this extra work to support the safety of their care and that the pandemic has just illuminated this issue. Future research should explore existing vulnerabilities and inequalities, and the heightened safety consequences created by the pandemic.

**Patient and Public Contribution:**

The National Institute for Health Research (NIHR) Yorkshire and Humber Patient Safety Translational Research Centre (NIHR Yorkshire and Humber PSTRC), Patient and Public Involvement and Engagement Research Fellow and NIHR Yorkshire and Humber PSTRC Patient Involvement in Patient Safety theme lay leader are involved in the preparation of a lay version of the findings within this manuscript.

## 1 INTRODUCTION

The resilience of a system is defined as its ability to maintain operations when there is a disruption.^1^ To do this, resilient systems are posited to be those able to respond, monitor, learn and anticipate^2^ in the face of organizational threats. To those responsible for health systems, the COVID‐19 pandemic can be categorized as an irregular threat that is unlikely to happen and is difficult to prepare for.^3^ The COVID‐19 pandemic has been a global health crisis which has demanded large‐scale population behaviour change. Globally, the daily lives of the general public were abruptly impacted in early 2020, with many countries instituting nationwide lockdowns in the interest of public safety. In April 2020, people across the United Kingdom were ordered to stay at home and leave only under certain circumstances. National Health Services (NHS) were suddenly reorganized, and resources were redirected to prepare for the COVID‐19 outbreak and treat increasing numbers of COVID‐19 patients. This had enormous implications for the way, both regular and irregular users of the NHS, experienced and accessed healthcare services, and for the quality and safety of care.^4^, ^5^

Given the large‐scale adaptations that have been required nationally and globally, the COVID‐19 pandemic in many ways represents a very visible, tangible expression of the concept of resilience,^6^ and one upon which it is useful to apply a resilient healthcare conceptual ‘lens’. Foundational to the concept of resilient healthcare, is that people acting within a system represent one of the main sources of adaptive capacity, and are thus key to the ability to maintain stable performance across variable conditions.^1^ Whilst staff within healthcare organizations have traditionally been seen as the main protagonists in this source of adaptive capacity,^7^ it is increasingly becoming recognized that patients and their families also contribute to the capacity of a system to adapt to fluctuating conditions.^8^, ^9^, ^10^ The ways in which patients and families might do this are many and varied. For example, patients and families have reported chasing appointments, following up on missed care and checking medications.^9^, ^10^, ^11^ This type of activity has come to be thought of as variously ‘scaffolding’ the healthcare system to support its ongoing performance,^7^ or ‘propping it up’ when care is suboptimal or there are significant structural ‘safety gaps’.^12^, ^13^

Whilst the role of patients and families in supporting their safety is increasingly being recognized and documented,^9^, ^10^, ^11^, ^13^, ^14^, ^15^ hitherto this supporting role has been limited to those directly ‘using’ services. However, given the enormous change to the fabric of society that was experienced in the early phases of the pandemic—often with the expressed purpose of ‘flattening’ the curve, or ‘saving lives’^16^—it is clear that members of the general public, including those who were not active users of health services during the COVID‐19 outbreak, were suddenly asked to undertake activity to support the NHS.

This paper presents the findings of a study within which we examined the experience of members of the public during the early phases of the COVID‐19 pandemic, within the United Kingdom. We aimed to understand what people were doing during the first wave of the pandemic to support the safety of their health and the health of others, and in doing so, if and how this may have supported the resilience of the healthcare system. To do this, we asked the following research questions:

(1) What are the public doing to individually and collectively support the safety of themselves, their immediate networks, society and healthcare systems?
(2) How did this change over time?

## 2 METHODS

### 2.1 Design, recruitment of participants and setting

This study used a qualitative interview method. The ontological paradigm of relativism was assumed, which suggests that researchers can never gain a fully objective access to the world since the world in an objective sense cannot be known. Using a postpositivist epistemological stance, researchers were, therefore, able to use the qualitative interview method to access, explore and gain insights into subjective human experiences, via a collaborative process mediated by human understanding.^17^ Smith and Osborn^18^,p.53 refer to this process as ‘the participants are trying to make sense of their world, the researcher is trying to make sense of the participants trying to make sense of their world’.

Participants were recruited via the social media platform Twitter. An invitation to participate was posted on Twitter, with recruitment proceeding using convenience sampling. Initial eligibility criteria were purposefully broad, including anyone over the age of 18 who could speak English. Those who expressed interest were emailed the information sheet, also available in an easy‐read format, and scheduled at a mutually convenient time for interview if they still wished to participate. However, once 10 participants were recruited, demographic information was reviewed and purposive sampling was conducted for the remaining sample to ensure diverse and representative views were captured.

Twenty‐one members of the public participated in 57 semistructured interviews over three time points from June to September 2020 (21 at time point 1, 19 at time point 2 and 17 at time point 3). The longitudinal nature of the interviews was novel as it allowed us to explore peoples' perceptions over time and in real‐time, rather than retrospectively. Interviews were conducted by two authors (A. A. and L. R.) using Zoom—an encrypted secure video conferencing software. Fifty‐one people expressed interest in taking part, 30 people were sent a holding email to facilitate purposive sampling and 21 people participated. All participants lived in the United Kingdom. Participant demographic information is displayed in Table 1.

**Table 1:** Participant demographic information

Participant pin	Age	Gender	Paid employment status	Ethnicity	Residential area
P1	74	Male	No—retired	White British	Rural
P2	69	Female	No—retired	White British	Suburban
P3	62	Female	No—retired	White British	Rural
P4	61	Female	Yes	White Australian	Suburban
P5	72	Male	No—retired	White British	Suburban
P6	55	Male	Yes	Asian British	Urban
P7	42	Female	No	Asian British	Urban
P8	44	Male	Yes	White British	Suburban
P9	56	Male	No	White British	Suburban
P10	46	Female	No	Asian British	Suburban
P11	30	Male	Yes	Black Caribbean	Suburban
P12	39	Male	Yes	White Polish	Suburban
P13	62	Male	Yes	Black British	Rural
P14	53	Female	No	Black	Suburban
P15	40	Male	Yes	Mixed race	Urban
P16	42	Female	No	Asian British	Urban
P17	19	Male	No—student	Asian British	Suburban
P18	66	Female	No—retired	White British	Urban
P19	37	Female	Yes	Asian British	Urban
P20	48	Female	Yes	White British	Urban
P21	20	Female	No—student	Arab	Suburban

### 2.2 Data collection procedure

Participants took part in an initial interview and were invited to two follow‐up interviews after 3 and 6 weeks. Participants could choose to be interviewed with their camera turned on or off, and their preference was mirrored by the interviewer. Verbal informed consent and demographic information were obtained. Separate recordings of consent and identifying information including the interview data were made. Audio recordings were transcribed verbatim. Participants each received a £20 voucher after the initial interview. A semistructured interview guide directed the conversation towards understanding the participants' background and use of health services, both prior to and during the pandemic. Follow‐up interviews focussed on any changes in the participant's experience, including emotions, attitudes and behaviours. Adaptations were made to the guide as interviews progressed so important topics were raised with subsequent participants.^19^ Initial interviews lasted between 27 and 81 min (mean of 50 min). Follow‐up interviews lasted between 14 and 62 min (mean of 30 min).

### 2.3 Analysis

Fifty‐five interview transcripts were included in the analysis. Our approach to analysis was principally inductive but organized around the sensitizing concept^20^ of patients, family or public supporting (‘scaffolding’) their health and healthcare service level outcomes. This approach allowed us to explore our findings with reference to the broad research objectives, whilst providing a point of reference to guide the analysis of the data.

A reflexive thematic analysis approach was used.^21^ Researchers have multidisciplinary expertise, with backgrounds in patient safety, psychology, qualitative methods and health services research. Four authors (A. A., L. R., I. H., C. G.) independently familiarized themselves with the transcripts. Data were initially analysed inductively, adopting a within‐person approach and producing in‐depth summaries of initial impressions for each participant across the time points. Relevant extracts were identified and brought together where they explored similar ideas to develop a preliminary coding framework. The in‐depth summaries were then organized according to the research questions and to the sensitizing concept of patient and public co‐creation of health and healthcare services.

The summary documents formed the unit of analysis for regular meetings between all authors, revisiting the raw data where necessary. The coding framework was discussed and refined until a consensus was reached. This included drawing upon the data set to gain a holistic view and highlighting significant extracts to define each theme to ensure that the analysis captured information relevant to the research questions, but also included novel perspectives. A detailed log of theme development was kept. The study followed the consolidated criteria for reporting qualitative (COREQ) research.^22^

## 3 RESULTS

The results are presented in three themes, each with its own subthemes. These are: (1) A ‘new safety normal’ (*Feeling cast adrift, Adapting to support and Balancing risks and unanticipated outcomes*); (2) Existing vulnerabilities and heightened safety (*Personal circumstances and safety ‘capital*’, *and Inequity of healthcare and information access*) and (3) Are we all in this together? (*Interpreting others' safety behaviours and motivations*, and *Government mistrust and its influence on public approaches to safety*).

### 3.1 A ‘new safety normal’

Sudden adaptations were made by health services in response to the COVID‐19 outbreak causing an enormous ripple effect which was felt in participants' daily lives. Simple, routine activities that were never viewed as safety‐related measures previously, were now considered via the lens of risk in efforts to reduce virus transmission. Individuals also referred to the continuous revaluation of both how they interacted with health services based on their perceived preparedness, and how they navigated potential threats posed to their safety.

#### 3.1.1 Feeling cast adrift

Some regular users of health services with complex physical and mental health needs described the impact that sudden changes in resources and services had on their care, including delays and the stopping of some services altogether. Others had concerns for particular groups, such as cancer patients, appearing to worsen existing vulnerabilities and resulting in those at most risk often being the least able to keep themselves safe. We perceived that regular users of health services, in particular, felt cast adrift, and left to independently monitor and make decisions. Participants discussed examples where they felt forced to step in and support their own care to ‘plug’ safety gaps both created and widened by the pandemic, such as chasing delayed tests, following up on results and coordinating care providers.

The rheumatology department got practically closed when Covid came along because all the staff got redeployed. So you were kinda left to have to start making your own decisions … she told me to go and see my GP … I rang 111 because they were saying don't go to your GP if you've got Covid … very conflicting advice. P16 (Interview 2)

Issues creating further difficulty for patients feeling cast adrift from the system included receiving conflicting information from health services, lacking personal protective equipment (PPE) and perceiving that digital interactions failed to meet their needs. For instance, one participant referred to providing carers who regularly visited prior to and during the pandemic with PPE, propping up the safety of themselves, care providers and others they came into contact with. Additionally, across settings, participants struggled to gain the same therapeutic reassurances of face‐to‐face care via remote alternatives. In a physical health context, one participant recalled attending Accident and Emergency to obtain face‐to‐face care, when they felt the remote support offered by their GP and physiotherapist was insufficient.

They [GP] weren't understanding, and you know, they said, ‘we don't think they'll see you for a face‐to‐face’, and I explained, ‘Look, I can't sleep on a night, I've not slept for five days, and exercise isn't going to work’. P13 (Interview 1)

In a mental health context, where participants did manage to access virtual support, they were initially reassured, but over time, felt that service adaptations were largely inadequate.

I've tried ringing her so many times, it's just diverted to her mobile. No‐one picks the phone up … [I had virtual advice] from the Mental Health Team, but it's the same information I suppose … again and again, yeah, just repeating themselves. P10 (Interview 3)

Face‐to‐face support was described as coming to an abrupt standstill by a number of participants who previously interacted with services regularly. People were left feeling alone in the absence of possible ‘lifelines’ when they potentially needed it most in the midst of the pandemic. Concerns centred on short staffing and poor continuity due to redeployment and illness. One participant who was a regular user of health services felt that their discharge from inpatient care was rushed and disorganized, and with the lack of community mental health support, was readmitted a month later. Despite difficulties accessing care, some did feel that other services were able to provide a safety net. For instance, general practice and acute hospital services were able to provide some mental health support.

I really didn't get on well with the kind of, new way of [remote] working … I'm suspicious of it, I don't think it works for me … My GP has really helped when the mental health services have let me down. P19 (Interview 1)

During the pandemic my specialist nurse got redeployed. So, I got another care coordinator … he just went off sick for three weeks. And so, my case was handed over to nobody … I wasn't feeling well anyway, the pandemic almost made it worse … when the lockdown was announced I was already [sectioned] inside. Then they quickly discharged me which was very unorganised and unprepared. P19 (Interview 1)

A participant working as bank staff at an assisted living facility for people with mental health problems sympathized with difficulties people were facing due to service adaptations, echoing the ineffectiveness of virtual support and the negative impact this was having on people's mental health and rates of readmission.

#### 3.1.2 Adapting to support

People made immediate behavioural adaptations to avoid overwhelming the NHS, resulting in a perceived demand reduction for healthcare services in the short term, decreasing variability in performance and creating more positive healthcare experiences for some. Participants described an initial improvement in the quality of healthcare they received during the first national lockdown, with a sense that services were overprepared at first. One of these participants described it as ‘the best service I have ever received’.

I'll have to say the last couple of weeks, months, massive improvement [in A&E]…. most importantly you become a person, before I didn't feel like they acknowledge you, I didn't feel like their mannerism was up to scratch, but from start to finish, I would absolutely say they acknowledge you and respect you as a person. P14 (Interview 1)

The adaptations people made were reflected in their views about the preparedness of the NHS and its ability to manage. Some had total faith in system resilience and perceived that the system was well‐equipped to deal with the ongoing challenges posed.

The NHS has almost over‐compensated for the worst possible scenario … the hospitals have got less people in than they expected … in terms of resources they're able to deal a lot more … I don't have any doubts in the NHS in that respect. P15 (Interview 1)

Interestingly, this view was mirrored by participants who were also healthcare staff. Adaptations made by both the system and society reduced short‐term demand, and healthcare staff participants commented on being able to spend time listening to patient concerns and providing quality care. Although the initial overpreparation subsided over time and the usual demand resumed, but was also superseded by an increased burden in terms of the workload intensity, care complexity and understaffing.

At the beginning it was wonderful … they were throwing staff at us and it was lovely … everything was getting done properly … the nurses had time to have lunch, to go to the toilet … Now the last few weeks we're back to how we were, there's no staff there's too many patients, it's full on again. P20 (Interview 1)

Many were particularly reluctant to access health services unless they perceived it to be absolutely necessary, due to both a perceived increase in the risk of contracting COVID‐19 and to help the NHS cope with the additional strain. Others were willing to continue as they normally would and didn't have concerns about their safety while using services in person. There was also a growing sense of confusion among participants about initiatives and approaches used by healthcare services in response to the COVID‐19 outbreak which limited their ability to make informed decisions, for instance, the use of Nightingale hospitals intended to treat the rising numbers of COVID‐19 patients.

What I don't understand is, we built all these Nightingale hospitals and we built them really quickly and well, but we never seemed to have used them. So why were normal hospitals taking in Covid patients when, in theory, the Nightingale hospitals were to take in Covid patients to allow normal patients to go to the hospitals? I've seen stories of, you know, people who've lost cancer treatments, people who've lost transplant treatments and transplant opportunities. P5 (Interview 1)

#### 3.1.3 Balancing risks and unanticipated outcomes

All participants expressed a continual trade‐off between protecting their physical and mental health. Engaging in behaviours to protect physical safety was sometimes to detriment to well‐being. For many participants, their original priority was protecting their physical safety by staying indoors and using virtual alternatives to socialise. As time progressed, the prioritization of physical safety lessened for some, and concerns for mental health grew fuelled by a sense of isolation, loneliness and monotony.

[I'm] pretty much staying in, mostly, until the end of this year, for sure, but I think, come next year I need to start thinking about going out … it's kind of just trying to find a way of balancing the risk of infection against the risk to our mental health of not doing normal things and socialising. P16 (Interview 1)

This shift in focus towards preserving mental health resulted in fluctuating willpower to follow guidelines designed to protect physical health. Some emphasized the importance of stepping away from information sources, particularly those considered unreliable, to reduce the sense of being inundated with threats to physical safety. The monitoring activity required to continually stay safe became too burdensome for some, who only consulted sources of information pertinent to them. Yet others who felt in a more physically vulnerable position described feeling unable to afford such luxury.

There was too much news. I thought if I want news I'll go and look for it. There were a lot of unnecessary stories … I was just getting a bit fed up with having the news whenever I went on there. P6 (Interview 2)

For instance, the imminent tightening of restrictions meant that some felt they should capitalize on having increased freedom, while others, such as those who were shielding, felt they needed to take additional precautions such as social selectivity.

My choir are finally having an in‐person meet up at the (local park), but I just decided not to go … they said ‘we can socially distance because we're in a big outdoor space, and bring your own food’ but then they said to share? [laughs] And I'm thinking, you do not really understand this transmission of the virus. P16 (Interview 3)

For some, the change to daily life was a largely positive experience personally, and they ‘admitted’ enjoying unexpected benefits. Although they expressed empathy for others in less fortunate situations, such as those who lived alone, who had limited access to outside space, whose livelihoods were affected, and who had lost loved ones. Those experiencing respite during lockdown tended to face difficulties before the COVID‐19 outbreak, such as caring duties, burnout from work or unmanageable social obligations. For these people, lockdown meant they were better able to focus on their own health and well‐being.

Personally, I have found lockdown to be a relaxation of a certain kind of pressure that I felt … I have always felt I must keep up, I must keep in touch, you know, I mustn't lose my friends, and to be told you can't actually go out and see them took a lot of pressure off me. P2 (Interview 1)

Some participants were contrastingly experiencing personal difficulties alongside managing threats to their physical and mental health exacerbated by the pandemic, such as bereavement and job insecurity.

I keep worrying about possible consequences, especially economic consequences … that worries me a lot because I don't know what will happen with my job. P12 (Interview 1)

### 3.2 Existing vulnerabilities and heightened safety consequences

For some, the ‘new safety normal’ was more familiar. People who had existing vulnerabilities were more likely to experience safety gaps in their care and be required to step in to scaffold their safety, despite it being more difficult for them to do so. The consequences of existing vulnerabilities and inequalities were exacerbated during the COVID‐19 outbreak. Different personal circumstances influenced peoples' chances of being able to protect themselves. Factors including income, disability, living and work arrangements impacted peoples' general safety and healthcare interactions. While there was an initial sense of community being fostered, over time, there were wider concerns about divisions in society developing throughout the pandemic.

#### 3.2.1 Personal circumstances and safety ‘capital’

The ability of people to ‘reach in’ into the system to support themselves was dependent on their capital and resources, meaning that some could support their own safety more easily than others. For instance, participants living in built‐up areas and those who didn't have a private vehicle felt trapped indoors, with walking through busy streets or using public transport as the only option to access health appointments, green space and leisure activities.

I haven't been on public transport in months and just going the one tube stop to go to this appointment for the bloods was sort of already a big deal … just getting to the train station, there's a lot of people on the road and they're all coming towards you and no one's social distancing. I'm constantly having to dodge people, it's quite stressful. P16 (Interview 2)

Several participants expressed concerns about returning to work, particularly in terms of how effectively their workplace would enforce social distancing and provide appropriate PPE. Those who could remain working from home felt better able to reduce their risk of contracting the virus.

I'm supposed to shield until the end of July so in August I will be going back to work and I think there's no option, the economy has to restart … I'd rather stay [home] a bit longer. P12 (Interview 1)

#### 3.2.2 Inequity of healthcare and information access

Inequities in healthcare and information access were identified, such as culture, language and clinical vulnerabilities providing barriers to supporting safety. Paradoxically, those deemed most vulnerable, such as those with pre‐existing health conditions asked to shield, were required to leave the safety of their homes to access healthcare services. Support designed to alleviate these issues, such as the volunteering initiative, was perceived to be ineffective by some. For instance, one participant was told that they needed to make their own transport arrangements, causing further stress.

They [volunteer service] actually told me, they said that you need to make your own plans to get there, in case no one's taking you … no one's contacting me, so I'm just assuming that no one's taking me so I've gotta get three trains … I avoid public transport, which as I said is really unavoidable now because I really have to go to the hospital next week. P16 (Interview 2)

A number of participants discussed the disproportionate effect of COVID‐19 on people from ethnic minorities and had personal connections with people from ethnic minorities who had died. The unequal provision of information and support was also highlighted, whereby government communication to people from ethnic minorities who had health conditions had been poor and was felt to be directly linked to the deaths of friends.

Not one of them was receiving any information from the government, or support, and they were all isolating bar one…. Only one gentleman, he was Caucasian, he died in his flat, but he was receiving food parcels, he was receiving support for his mental health, but all the others wasn't receiving anything, and they were all ethnic minorities. So that does concern me. P14 (Interview 1)

### 3.3 Are we all in this together?

There was a general sense of empathy for how difficult others' circumstances were during the pandemic, contributing to societal efforts to ‘flatten the curve’. Differences in peoples' approaches to risk and safety were highlighted over time. Perceptions of the government's response to the virus appeared to directly impact peoples' safety behaviours, resulting in many using their own initiative rather than following government guidance.

#### 3.3.1 Interpreting others' safety behaviours and motivations

Participants became aware that others' behaviour could be a danger to their own safety and paid attention to others' safety behaviours and motivations because there was suddenly a collective risk to health. Most participants acknowledged that their approach and behavioural adaptations were not shared by everyone. Some felt that this was a result of a lack of understanding or disregard for the rules. A few participants felt it was young people who were not using common sense and causing the virus to spread, a perspective that links to the development of divisions in society during the pandemic.

Society seems to be splitting into those, say, under thirty who seem to believe that they can go out, they can go to raves, they can go to parties, and those other who are saying, oh, well, we'll go out and we'll be sensible and we'll think about things. P5 (Interview 2)

Participants tended to have the view that they wanted to ‘do their bit’ and make a positive contribution to society by following government guidance, but also taking extra precautions they felt necessary. This was with a view that others ought to do the same to be beneficial at a societal level. However, some considered that this view was not shared by others, who acted against guidance, albeit understandably, rather than pulling together for the greater good.

You see all this queuing at Primark and I'm just thinking … why? But I think there is something more than just going shopping, it's actually a day out for some people. 'Cause lockdown is not easy, we are social creatures. P3 (Interview 1)

#### 3.3.2 Government mistrust and its influence on public approaches to safety

While personal perceptions of the government's response to the virus varied, many felt that there had been a shift in emphasis from what was saving lives and protecting the NHS to protecting the economy. A small number of participants who were more mistrustful of the government felt they were acting disingenuously. On the other hand, many individuals spoke about ‘muddling through’ with the sense that nobody really knew what the best approach was, even those setting the guidance.

I think that's the difficult thing about everything, isn't it, we don't know, and even the government, they don't know, they've never dealt with it before. We're all just really trying to go along in the dark, aren't we blindly. P20 (Interview 2)

For those finding the government guidelines to be particularly ambiguous, there was an increasing responsibility to behave according to their own initiative. Examples of the government not following the guidelines it set out fuelled government mistrust and resulted in participants taking greater personal responsibility for their decisions. In the perceived absence of trustworthy information, many participants referred to using ‘common sense’, whether that be to impose stricter behavioural adaptations, or to interpret the rules in a way they deemed logical. This included those who were shielding and who were anxious about the easing of measures for economic reasons.

I generally find that everyone I know who is shielding is saying ‘I'm gonna make my own decision and I'll go out and do what I think is safe and not trust at all in what the government are now saying is suddenly safe’. P16 (Interview 2)

Guidance was in some cases adapted to suit their circumstances, for example, bubbling two households of more than one person. Other participants interpreted guidance with a focus on maintaining as much normalcy as possible for themselves and their families. This was particularly pertinent for one participant who cared for a child with autism.

Since it's eased, we've allowed my son to play outside with his friends, and erm, I've tried to keep in touch with parents, online texting and emailing … I think I've made the effort because, not 'cause I feel the need socially, but I think this crisis is gonna go on and on and on, if I'm not careful we'll lose all our contacts, which doesn't worry me so much 'cause I'm not a particularly sociable person but I've built a lot of contacts for my son because the social aspect of autism is critical. P9 (Interview 1)

## 4 DISCUSSION

This study sought to understand what people were doing during the first wave of the pandemic to support the safety of their health, the health of others and the resilience of the healthcare system. To do this, we explored the experiences of members of the public across the first wave in the United Kingdom, regardless of the regularity of their engagement with the NHS/other inclusion criteria often used in COVID‐19‐related research. We found that almost universally, participants made adaptations to their daily lives that whilst disruptive to them, was done to support the safety of themselves, their immediate families and communities and the NHS. Re‐organization of healthcare services and society had a significant impact on people's lives. Some who used healthcare services regularly felt abandoned by the healthcare system and needed to undertake significant roles in supporting their own care, for example, chasing up appointments and follow‐ups. We also found that the behavioural adaptations made by some to protect the healthcare system by avoiding overwhelming the NHS resulted in others perceiving better care. Our findings pointed to differences in people's approaches to risk and safety and that those approaches changed over time to protect their mental well‐being.

### 4.1 Was there evidence of a ‘scaffolding’ role?

Resilient healthcare approaches and theories have for a long time regarded staff working in healthcare systems as a flexible and adaptable resource that contributes to its ability to cope with variable conditions and unexpected events.^1^ Building on this, a number of studies have begun to describe how patients and families have a significant and often underrecognized role in the safety of healthcare services and systems and that their roles are a source of resilience in system performance.^9^, ^10^, ^23^ This type of activity has been conceptualized as patients and families ‘reaching in’ to healthcare systems to bolster the safety of its activity.^7^ To put it another way, it might be argued that this activity acts as a ‘scaffold’ of the healthcare system, by providing an extra layer of adaptation to reduce, or ‘dampen’ its performance variability.^7^ This activity has also been described by other authors as ‘propping up’ to the safety of healthcare systems through the actions that they take to bolster safety.^12^ However, whilst this ‘buffering role’ is often provided by patients and their families, the pandemic has shined a new and important light on this issue. Our findings suggest that the activity of the public can, and should be conceived as being part of the wider resource (previously conceived as staff, and now staff, patients and families) that supports the ability of the healthcare system to adapt when faced with sudden, irregular threats.^3^ Indeed, it is also likely to be true during ‘normal conditions’. In some of her early explorations of the workings of public services, Elinor Öström described how the ‘success’ of police services can be in part attributed to communities, who effectively ‘co‐create’ crime statistics, by the activities they undertake (e.g., phoning in suspicious characters, or getting locks upgraded).^24^ As such, it is not just what victims of crime are affected by, or affect the crime statistics, but all community members. Our findings suggest that this is also a useful way of conceiving the role of citizens in relation to the outcomes of healthcare services.

### 4.2 Was everyone in it together?

It is clear that whilst all participants made adaptations, their impact was unequally felt. Study participants—members of the general public that included both NHS patients and staff—described how they would not use services unless it was absolutely necessary, thereby protecting the NHS from becoming overwhelmed. However, it was those who perceived a greater threat and were most at risk that needed to undertake the most work to keep themselves safe. For others, the service stopped completely. These findings resonate with other literature which has demonstrated inequity in the impact of the pandemic. For example, more deprived areas and those with higher non‐White populations have been found to have higher mortality rates in the early stages of the pandemic.^25^, ^26^ Our findings raise a profound paradox in the necessary adaptations that everyone had to make during the pandemic—that those who needed services the most, had to make the biggest adaptations to their lives to keep themselves safe, and their health was put most at risk. Put simply, our findings build on other evidence that the existing structural inequalities in society were highlighted and exacerbated by the pandemic.^25^, ^27^, ^28^

### 4.3 The adaptation of adaptations over time

Our findings also highlight an interesting issue about the impact of adaptation longevity on system resilience. Our participants described how across the course of the first wave, they experienced a growing burden associated with monitoring their own and others' safety. As the pandemic progressed, they articulated the ‘trade‐offs’ they made between keeping physically safe and mentally well. Some described protecting themselves from the burden of this monitoring activity, by taking a break from seeking out or listening to information sources about COVID‐19. This finding is important, as to be effective, any crisis response must engage with the public. Such engagement can be done using different elements, including transparency, ethical reasoning and formal and informal deliberation.^29^ Official information sources in England mainly used transparency elements, focusing on daily briefings and local healthcare systems information releases. Our study shows that people's tolerance for such information is finite and that different engagement mechanisms should be employed to support ongoing crisis responses. Such a finding is supported by a recent literature review exploring the nature of effective pandemic communication.^6^

There was also some evidence that as time progressed, people saw others violating the rules, including prominent public figures, and so the credibility of the public messaging experienced attrition. Researchers have characterized the impact of the pandemic, and evidence that others had not followed the rules around isolation, as being morally injurious.^29^ They argue that efforts to limit the spread required people to prioritize the safety of others over their own moral commitments to friends and family, for example, seeing and visiting relatives and attending funerals.^29^ Whilst our participants did not describe their own experiences as being morally injurious, the impact of isolating and monitoring information to keep safe impacted their mental well‐being.

### 4.4 Limitations

It should be noted that social media (Twitter) was used to recruit participants for the study. Although Twitter does have the ability for social reach and was felt to be an appropriate method of recruitment given the limitations created by the pandemic, this may have biased the sample to include only those who use social media. Using a different method of recruitment that did not rely on social media may have resulted in a sample of people with different perspectives than those reported. Finally, we were not able to follow up with all participants across all time points, which might have impacted the inferences from our study relating to changes in experiences over time.

## 5 CONCLUSION

Our study exploring how the public co‐created health and healthcare safety during the COVID‐19 pandemic, found that they had an important role in supporting the resilience of healthcare services and systems during the first wave of the pandemic. This was evident through significant adaptions to their behaviour, explicitly undertaken to protect themselves and others and to avoid overwhelming the NHS. Our findings illuminate existing inequalities and inequities for those who are most vulnerable, and who were already required to safely navigate inadequacies or ‘gaps’ in their healthcare. These ‘safety gaps’ may have widened during the COVID‐19 pandemic, leading to a paradoxical situation where the most vulnerable were required to undertake the most ‘scaffolding’ activity. Our findings can also usefully be understood within the context of earlier work on the success of public services,^24^ whereby all citizens, not just users of the services, contribute to individual and system‐level outcomes.

## AUTHOR CONTRIBUTIONS

Jane K. O'Hara brought the initial research idea to the team. Abigail Albutt and Lauren Ramsey conducted the interviews. Alongside an outsourcing company, Isabel Hague and Chloe Grindey transcribed the interviews. All authors discussed and refined the coding framework. Abigail Albutt, Lauren Ramsey, Isabel Hague and Chloe Grindey applied the agreed coding framework and drafted the analysis for individual participants and each concept. Abigail Albutt and Jane K. O'Hara drafted the introduction. Abigail Albutt and Lauren Ramsey drafted the methods and analysis reflecting on the whole data set. Beth Fylan and Jane K. O'Hara drafted the discussion. All authors commented on, refined and iterated the paper.

## CONFLICT OF INTEREST

The authors declare that there is no conflict of interest.

### ETHICS STATEMENT

Ethical approval was granted by the University of Leeds School of Medicine Ethics Committee (MREC 19‐076).

## Supporting information

Supporting information.

## Data Availability

The data that support the findings of this study are available from the corresponding author upon reasonable request.
