# Supplementary material for: Patient and public co‐creation of healthcare safety and healthcare system resilience: The case of COVID‐19
Source: Health Expect. 2023 May 4;26(4):1467–77. doi: 10.1111/hex.13659 (PMC10349237; doi:10.1111/hex.13659)
Supplement: Supplementary file 1 — Supporting information. [file HEX-26--s001.doc]

**Consolidated criteria for reporting qualitative studies (COREQ): 32-item checklist**

Developed from: Tong A, Sainsbury P, Craig J. Consolidated criteria for reporting qualitative research (COREQ): a 32-item checklist for interviews and focus groups. International Journal for Quality in Health Care. 2007. Volume 19, Number 6: pp. 349 – 357

| **Domain 1: Research team and reflexivity** |  | **Reported on page no.** |
| --- | --- | --- |
| **Personal characteristics** |  |  |
| 1. Interviewer/facilitator   Which author/s conducted the interview or focus group? | AA, LR | 6 |
| 1. Credentials   What were the researcher’s credentials? E.g. PhD, MD | PhD | - |
| 1. Occupation   What was their occupation at the time of the study? | Research Fellow | - |
| 1. Gender   Was the researcher male or female? | Female | - |
| 1. Experience and training   What experience or training did the researcher have? | The researchers were  experienced academic  researchers educated to PhD  level, AA and LR (both female)  research patient safety. | - |
| **Relationship with participants** |  |  |
| 1. Relationship established   Was a relationship established prior to study commencement? | No | - |
| 1. Participant knowledge of the interviewer What did the participants know about the researcher? e.g. personal goals, reasons for doing the research | No prior knowledge. | - |
| 1. Interviewer characteristics   What characteristics were reported about the interviewer/facilitator? e.g. Bias, assumptions, reasons and interests in the research topic | This is covered on page 7. | 7 |
| **Domain 2: Study design** |  |  |
| **Theoretical framework** |  |  |
| 1. Methodological orientation and Theory   What methodological orientation was stated to underpin the study? e.g. grounded theory, discourse analysis, ethnography, phenomenology, content analysis | A reflexive thematic analysis  approach was used. | 7 |
| **Participant selection** |  |  |
| 1. Sampling   How were participants selected? | The method of recruitment was  via social media, specifically  Twitter. An invitation to  participate was posted on  Twitter, with recruitment  proceeding using convenience sampling. | 6 |
| 1. Method of approach   How were participants approached? e.g. face-to-face, telephone, mail, email | Email | 6 |
| 1. Sample size   How many participants were in the study? | Twenty-one | 7 |
| 1. Non-participation   How many people refused to participate or dropped out? Reasons? | It was not known reasons why did not participate. | - |
| **Setting** |  |  |
| 1. Setting of data collection   Where was the data collected? e.g. home, clinic, workplace | Data was collected via zoom  from AA and LR’s homes. | - |
| 1. Presence of non-participants   Was anyone else present besides the participants and researchers? | No | - |
| 1. Description of sample   What are the important characteristics of the sample? e.g. demographic data, date | Described in Table 1. | Table 1. |
| **Data collection** |  |  |
| 1. Interview guide   Were questions, prompts, guides provided by the authors? Was it pilot tested? | Interviews were semi-structured  and a topic guide was used. | 6 |
| 1. Repeat interviews   Were repeat interviews carried out? If yes, how many? | Yes, data were collected at 3 time points. | 6 |
| 1. Audio/visual recording   Did the research use audio or visual recording to collect the data? | The interviews were recorded  using visual or audio recordings  depending on participants’  preference. | 6 |
| 1. Field notes   Were field notes made during and/or after the interview or focus group? | No, interviews were transcribed. | - |
| 1. Duration   What was the duration of the interviews or focus group? | Initial interviews lasted between  27 and 81 minutes (mean of 50  minutes). Follow-up interviews  lasted between 14 and 62  minutes (mean of 30 minutes). | 7 |
| 1. Data saturation   Was data saturation discussed? | No | - |
| 1. Transcripts returned   Were transcripts returned to participants for comment and/or correction? | No | - |
| **Domain 3: Analysis and findings** |  |  |
| **Data analysis** |  |  |
| 1. Number of data coders   How many data coders coded the data? | Four | 7 |
| 1. Description of the coding tree   Did authors provide a description of the coding tree? | No | - |
| 1. Derivation of themes   Were themes identified in advance or derived from the data? | Our approach to analysis was  inductive, given the broad nature  of the research questions. A  reflexive thematic analysis  approach was used. | 7 |
| 1. Software   What software, if applicable, was used to manage the data? | NA. | - |
| 1. Participant checking   Did participants provide feedback on the findings? | No | - |
| **Reporting** |  |  |
| 1. Quotations presented   Were participant quotations presented to illustrate the themes / findings? Was each quotation identified? e.g. participant number | Yes, participant quotations were presented to illustrate findings. Each direct quote was attributed to anonymised participant number. | 7-16 |
| 1. Data and findings consistent   Was there consistency between the data presented and the findings? | Yes | - |
| 1. Clarity of major themes   Were major themes clearly presented in the findings? | Yes | 7-16 |
| 1. Clarity of minor themes   Is there a description of diverse cases or discussion of minor themes? | Yes | 7-16 |
